# The Amazing Power of Cancer Cells to Recapitulate Extraembryonic Functions: The Cuckoo's Tricks

**DOI:** 10.1155/2012/521284

**Published:** 2011-09-28

**Authors:** Jose-Ignacio Arias, Maria-Angeles Aller, Isabel Prieto, Ana Arias, Zoe de Julian, Heping Yang, Jaime Arias

**Affiliations:** ^1^General Surgery Unit, Monte Naranco Hospital, 33012 Oviedo, Asturias, Spain; ^2^Surgery I Department, School of Medicine, Complutense University of Madrid, 28040 Madrid, Spain; ^3^Surgery Department, La Paz Hospital, Autonomous University of Madrid, 28046 Madrid, Spain; ^4^Internal Medicine Department, Puerta de Hierro Hospital, Autonoma University of Madrid, Majadahonda, 28013 Madrid, Spain; ^5^General Surgery Unit, Virgen de la Salud Hospital, 45004 Toledo, Spain; ^6^Division of Gastroenterology and Liver Diseases, USC Research Center for Liver Diseases, Keck School of Medicine USC, Los Angeles, CA 90033, USA

## Abstract

Inflammation is implicated in tumor development, invasion, and metastasis. Hence, it has been suggested that common cellular and molecular mechanisms are activated in wound repair and in cancer development. In addition, it has been previously proposed that the inflammatory response, which is associated with the wound healing process, could recapitulate ontogeny through the reexpression of the extraembryonic, that is, amniotic and vitelline, functions in the interstitial space of the injured tissue. If so, the use of inflammation by the cancer-initiating cell can also be supported in the ability to reacquire extraembryonic functional axes for tumor development, invasion, and metastasis. Thus, the diverse components of the tumor microenvironment could represent the overlapping reexpression of amniotic and vitelline functions. These functions would favor a gastrulation-like process, that is, the creation of a reactive stroma in which fibrogenesis and angiogenesis stand out.

## 1. Introduction

In the nineteenth century, Rudolf Virchow postulated a link between cancer and inflammation on the basis of observations that tumors often arose at sites of chronic inflammation, and the inflammatory cells were present in biopsied samples from tumors [1]. Twenty-five years ago, Dvorak recognized that the composition of tumor stroma is very similar to that of granulation tissue of healing skin wounds. He therefore suggested that “tumors are wounds that do not heal” [2]. Since that time, inflammation has been implicated in tumor development, invasion, and metastasis and in the development of clinical features as fever and cachexia [3]. These observations suggested that common cellular and molecular mechanisms are active in wounds and in cancer tissue [4].

## 2. Wound Repair Phenotypes: Gastrulation Revisited

The inflammatory response that is induced in the injured skin could be described as a succession of three overlapping phases during which the phenotypes of metabolic progressive complexity in using oxygen are expressed. Each one of these phases emphasizes the trophic role of the mechanisms developed in the damaged tissue. Hence, nutrition by diffusion predominates in the first phase; trophism is mediated by inflammatory cells in the second phase and finally blood circulation and oxidative metabolism play the most significant nutritive role in the third phase [5].

In the first or immediate phase of the inflammatory response, interstitial hydroelectrolytic alterations stand out. The vasomotor response, with vasoconstriction and vasodilation, is responsible for the ischemia-reperfusion phenomenon, which in turn causes oxidative and nitrosative stress in the injured tissue. In this phase, during the progression of the interstitial edema, the lymphatic circulation is simultaneously activated. In the following intermediate phase of the inflammatory response, the tissues are infiltrated by inflammatory blood-born cells, particularly leukocytes. Symbiosis of the leukocytes and bacteria for extracellular digestion by enzyme release, that is, phagocytosis, produces enzymatic stress. Furthermore, macrophages and dendritic cells take advantage of the lymphatic circulation activation and migrate through it until reaching the lymph nodes, where they activate lymphocytes [5, 6].

During the third phase of the inflammatory response, angiogenesis permits numerous substances, including hormones, to be transported by the blood circulation. Although the final objective of angiogenesis is to form new mature vessels for oxygen, substrates, and blood cell transport, other functions could be carried out before the new mature vessels are formed. Thus, angiogenesis could have antioxidant and antienzymatic properties, favoring therefore the resolution of the inflammation, as well as wound repair by epithelial regeneration and scarring. Consequently, in this phase the new formed tissue is structured, specialized, and matured by remodeling [5–7] (Table 1). 

 It has been previously proposed that the inflammatory response associated with the wound healing process in the skin could recapitulate ontogeny through the reexpression of two hypothetical embryonic trophic axes, that is, amniotic and yolk sac or vitelline, in the interstitial space of the injured tissue. If so, inflammation could represent the debut during postnatal life of ancestral biochemical mechanisms that were used for normal embryonic development. The re-expression of these ancient mechanisms, with a prenatal solvent path, is perhaps inappropriate and hard to recognize since they are anachronistic during postnatal life and because they are established in a different environmental medium [8].

**Figure 1:**
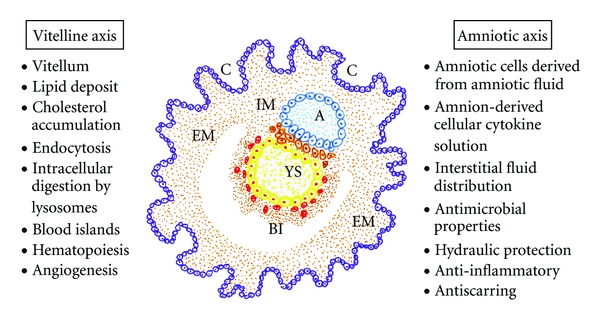
*Representative drawing of the early mammalian embryo during gastrulation*. The internalization of the extraembryonic mesoderm (EM) during gastrulation allows for the creation of the intraembryonic mesoderm (IM) that could thus join functional amniotic and vitelline properties from the amnion (A) and yolk sac (YS), respectively. BI: blood islands; C: chorionic vellosities.

The molecular and cellular contribution made by the two extraembryonic tissues that surround the fetus, the amnion and yolk sac, to the interstitial space located between them, namely, the mesoderm, are essential for organogenesis [9]. It could be assumed that both cavities are controlled by an array of inductive and inhibitory signals originating from the adjacent extraembryonic mesenchyma [8] (Figure 1). 

The amniotic axis could play a leading role in primitive interstitial hydroelectrolytic changes. The early mammalian embryo already has the ability to manage fluids in the interstitial space. Body fluid is distributed among three major fluid spaces, that is, intracellular fluid, interstitial fluid, and plasma. Nevertheless, the fluid distribution in each of these compartments is dramatically different in the fetus compared to the adult. Particularly, the amniotic fluid that surrounds the fetus may be considered an extension of the extracellular space of the fetus. Thus, the lymphatic system plays an essential role in the regulation of fluid distribution between the plasma and the interstitial fluid and, probably, with the amniotic fluid [10]. In addition to the rich amino acid content, amniotic fluid contains abundant peptides, carbohydrates, lipids, hormones, and electrolytes with water [11]. The amniotic fluid has antimicrobial properties, and this may be part of the innate immune system. A reduced volume of amniotic fluid may decrease the natural host defense conferred by this fluid and predispose it toward intrauterine infections [12]. Finally, it has been shown that amniotic-fluid-derived stem cells are able to differentiate into neurogenic, mesodermal, and endodermal lineages [13].

The vitelline axis, represented by the yolk sac, is the final destination of migrating visceral endoderm cells. The visceral yolk sac expands, and blood islands, structures consisting of hematopoietic progenitors surrounded by a loose network of endothelial cells, appear [14]. Endothelial cell precursors associated with blood islands differentiate and coalesce to form a primitive circulatory bed, which later connects to the embryo via the vitelline vessels [15]. Also, a major function of the yolk sac is the accumulation of carbohydrates, proteins, and lipids (*vitellum*) for embryo nutrition [16, 17]. Particularly, the yolk sac plays a vital role in providing lipids and lipid-soluble nutrients to embryos during the early phases of development [16, 18]. The yolk sac uses high-density lipoproteins (HDLs) and very-low-density lipoproteins (VLDL) as carriers to incorporate cholesterol from the maternal circulation and to transfer it to the embryonic side [16]. In turn, the interstitial lipid accumulation of cholesterol, a precursor molecule of many hormones like aldosterone, corticoids, androgens, strogens, and progesterone, may favor fluid infiltration and cell migration, proliferation, and differentiation during embryo development [19]. The ability to transport fat in the form of lipoprotein through the circulatory system by eukaryotes is one of their most significant functions right from the beginning of existence [20].

It could be accepted that these primitive functions are internalized during gastrulation to create the intraembryonic mesoderm. Thus, this germ layer would integrate the amnion- and yolk-sac-related functions [8]. Fibroblasts are mesodermal derived cells, and perhaps this embryonic origin could justify their great postnatal plasticity. The mesodermal cells of the embryo participate in the extraembryonic structures, including the chorion, the amnion, and the yolk sac [9]. In the human body, the fibroblasts form a heterogeneous collection of mesenchymal cells and they are the principal cellular constituents of connective tissues [21]. The major role of the mesodermal cells and their ability to differentiate from the first stages of embryonic development allow for considering them as the cell prototype that should be resorted to when the repair of any tissue in the body is needed [9]. And for this reason, perhaps the posttraumatic inflammatory response after a skin wound has the same intention, namely, to use the embryonic mesodermal phenotype with a therapeutic objective [8].

Local fibroblasts residing in the skin are considered the most prominent source of myofibroblasts. However, a variety of other precursor cells contribute to the myofibroblast population, depending on the nature of the injured tissue and the particular microenvironment [22]. Activated myofibroblasts are generated from a variety of sources, including resident mesenchymal cells, epithelial and endothelial cells, via the epithelial/endothelial mesenchymal transition as well as from circulating fibroblast-like cells called fibrocytes, derived from bone marrow stem cells [21, 23]. In addition, circulating monocytes have the capacity to differentiate into nonphagocytes, that is, mesenchymal cells and endothelial cells [24].

The reason why the myofibroblasts are attractive to a broad scientific and clinical audience is due to the large panel of cells that can develop this phenotype upon activation. It appears that myofibroblasts can be recruited from any local cell type and they are suitable for rapidly repairing injured tissue [22]. Fibroblasts can be induced to acquire the myofibroblast phenotype during wound repair. Several days after injury, a subset of wound fibroblasts can differentiate into myofibroblasts, which are responsible for repopulating the wounded area in parallel to angiogenesis, thus forming the granulation tissue [6, 7]. Granulation tissue is then repopulated with fibroblasts to produce a more densely collagenous extracellular matrix which is more akin to the matrix found in interstitial stroma [25].

Tissue remodeling requires the removal of granulation tissue, and maturation of collagen is oxygen dependent [6]. Indeed increasing wound oxygenation results in increased collagen deposition and tensile strength [26]. However, hypoxia benefits the expansion, differentiation, adhesion, growth factor secretion, and regenerative potential of mesenchymal stem cells derived from subcutaneous adipose tissue [27]. Finally, the phenotypic changes suffered by the keratinocytes during reepithelization suggest a partial epithelial-mesenchymal transition. Following the completion of wound repair, keratinocytes revert from their mesenchymal-like phenotype to an epithelial phenotype [28, 29].

In the adult organism, many pathways that play an essential role during embryo development are inactivated later in life although some of them may be transiently expressed during adult repair [30]. That is why we have considered that wound repair would require the upregulation of signaling pathways characteristic of the extraembryonic functions, that is, amniotic and vitelline, during the embryo development. If so, emulation by the wound tissue of these extraembryonic functions perhaps requires retracing the mechanisms that produce and distribute the extracellular fluid and substrates; activate the migration and invasion of stem or progenitor cells and hematopoietic-derived cells, and induce the establishment of a complex vascular network by lymphangiogenesis and angiogenesis; finally all of them are required to mediate normal new tissue growth and development [8]. The above-mentioned extraembryonic mechanisms seem to be aimed at favoring the grafting of new repaired tissue. Thus, through the successive expression of functions related to natural immunity and later with the acquired immunity, the neoformed tissue is accepted by the host.

This transient recapitulation of embryonic mechanisms through the hypothetical succession of overlapped amniotic-like and vitelline-like functions would achieve the cellular and metabolic diversity necessary for repairing the injured adult tissue with a graft (Table 1).

## 3. The Inflammatory Cancer Cell

Nowadays, the causal relationship between inflammation and cancer is widely accepted [1, 3, 4, 31–37]. Inflammation has long been thought to have contributed to the development of cancer [1, 35, 37]. And consequently, chronic inflammation is a major cause of cancer [32, 34, 36].

The understanding of the pathogenesis and progression of cancer requires the establishment of the altered genetic/metabolic factors that are essential to the development, growth, and proliferation of malignant cells [38]. This new frontier of cancer research requires the appropriate marriage of genetic/proteomic studies or the geneticist approach to the biochemical/metabolic cellular studies or the biochemical approach [38].

With regards to the geneticist approach, in many cancers a stem cell tumor model probably takes place [39]. Although a stem cell may sustain the first oncogenic hit, subsequent alterations required for the genesis of a cancer stem cell can occur in descendent cells [40]. This mechanism could explain the marked tumoral heterogeneity, either interturmoral, that is, variability between tumors arising in the same organ, or intratumoral, that is, variability within individual tumors [40].

With regards to the biochemist approach, metabolic transformation of malignant cells is essential to the development and progression of all cancers [38, 41–43]. Cancer cells, similar to normal cells, live in niches and microenvironments that are heterogeneous. Specifically, intratumoral gradients of nutrients and oxygen could play a profound role in modulating tumor cell metabolism [42].

**Figure 2:**
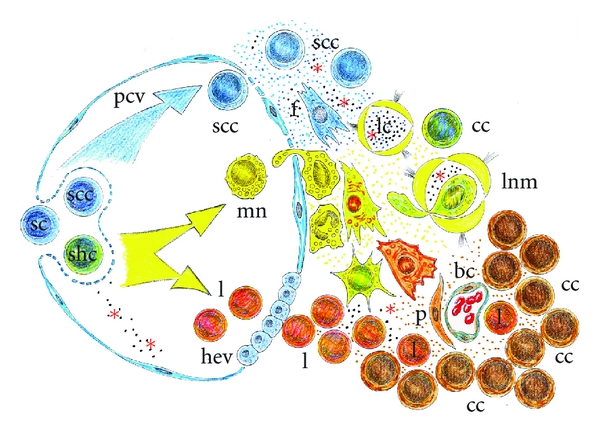
*Evolutive phases of the inflammatory cancer cell.* Cancer cells can adopt an inflammatory phenotype to invade neighboring tissues and survive in these ectopic sites. In the successive phases of tumorigenesis, the cancer cells invade the host by expressing natural and adaptive immune-related mechanisms. sc: stem cell; scc: stem cancer cell; shc: stem hematopoietic cell; f: fibroblast; mn: monocyte; mf: myofibroblast; g: granulocyte; mØ: macrophage; lc: lymphatic capillary; cc: cancer cell; lnm: lymph node metastasis; hev: high endothelial venule; l: lymphocytes; pcv: postcapillary venule; bc: blood capillary; p: pericyte; *tumoral antigen.

The comparison of tumors with wounds that do not heal [2] suggests that during the host invasion, the malignant tumor cells could express the previously proposed inflammatory phenotypes during wound repair [44]. Due to the plasticity of cancer stem cells, it should be kept in mind that while a malignant tumor develops, it can express phenotypes that also share the inflammatory response such as an ischemic phenotype (hypoxic) with edema and lymphangiogenesis (circulatory switch) a leukocytic phenotype being adapted, with migration to the regional lymph nodes and development of cachexia, and an angiogenic phenotype with the supply of nutrients and oxygen and tumoral mass growth [44–46] (Figure 2). 

It has already been proposed that these phenotypes represent the expression of trophic functional systems of increasing metabolic complexity in the wound inflammatory response [5, 6]. Their expression by cancer cells could have a similar significance [46]. In this hypothetical circumstance, malignant tumor cells could adopt an inflammatory-like phenotype that evolves in three hypothetical functional phases of increasing metabolic complexity and which would also have a trophic significance [44–46].

Moreover, acute wounds are initially hypoxic, and chronic ischemic wounds are essentially hypoxic [47, 48]. However, in the wounded tissue molecular and functional heterogeneity could be related to the heterogeneous distribution of oxygen with hypothetical pockets of graded levels of hypoxia [48].

The hypothesized similarity of the inflammatory response in wounds and tumors, based on the molecular-related, that is, genetic and metabolic, pathways is an interesting proposal because it could allow for translational inflammatory research between tissue repair and cancer. Moreover, the cancer cell also successively uses the natural and acquired immunity to be grafted into the host. The need to be recognized by the host, by both the cells that make the wound repair and by the cells that cause tumorigenesis to develop, would indicate that in the adult organism the creation of a new tissue without a previous immunologic acceptation could not be possible. Regardless of whether the neoformed tissue is normal, like during embrionary development and wound repair, or pathological, like in cancer, the expression of extraembryonic functions would be essential for homing in the host. Therefore, maybe the different inflammatory-related extraembryonic ways that converge in the embryo, in the wound repair process and in tumor growth, have the final aim of getting their immunological acceptation. 

## 4. The Evolutive Phases of the Inflammatory Cancer Cell

It has been proposed that the relation between inflammation and tumor development depends on whether the tumor is benign or malignant [44]. The benign tumor cells seem to be able to induce the inflammatory response in the host. Therefore, it is possible that the host participates in establishing the tumor through a process called desmoplasia, which consists of fibroblastic cells and the extracellular matrix, a leukocytic response represented by lymphocytes, macrophages, and dendritic cells, and lymphangiogenesis and angiogenesis [49]. Essentially, all the elements that constitute the inflammatory response participate in the “host reaction,” which could, therefore, have a trophic purpose for the tumor cells [44, 46]. Consequently, this inflammatory response of the host would develop in the interstitial space of the tumor, which invades in order to be more efficient, trophically [46].

However, when tumor cells reach higher grades of malignancy, their invasive capacity reflected in the classic metastatic cascade, that is, primary tumor (T), lymph node invasion (N), and distant metastases (M), seems to reflect a new capacity to express the inflammatory response more than to induce it in the host [45]. In this hypothetical circumstance the inflammatory phenotypes would be expressed by tumor cells to invade the host.

Therefore, it should be kept in mind that while a malignant tumor develops it can express phenotypes that are common to the inflammatory response, including a hypoxic phenotype with edema and lymphangiogenesis, a leukocytic phenotype with migration to the regional lymph nodes and distant metastasis, and an angiogenic phenotype with granulation tissue development and tumor and metastasis growth.

### 4.1. The Hypoxic Phenotype and the Interstitial-Lymphatic Tumoral Axis

Hypoxia and inflammation meet at several points in the setting of cancer [50]. During the initial avascular stages of tumor growth, which is when the tumor mass measures less than 0.5 cm [51], the cells seem to adopt an anoxic-hypoxic phenotype [52]. When tumor cells come into contact with oxygen, they could undergo a process of reoxygenation, with oxidative stress and edema [44].

The experimental evidence indicates that the cell mechanism for adapting to hypoxia is the prolyl-hydroxylase- (PHD)-hypoxia-inducible transcription factor (HIF) system [50]. The distribution of PHD-HIF system within the inflamed tumor is involved in both its growth and vascularization [50]. In this way, cancer cells could adopt a hypoxic metabolism to survive. Most cancer cells rely on aerobic glycolysis, a phenomenon termed “the Warburg effect.” Thus, cancer cells can convert glucose and glutamine into biomass most efficiently and will proliferate faster [53]. In addition, glutamine metabolism has important “nonanabolic” functions, including the regulation of oxidative stress, signal transduction, and autophagy [43].

Cancer cells at this stage can overexpress matrix metalloproteinases (MMPs). In many instances, therefore, the extensive alterations produced by MMPs in the stromal microenvironment could promote tumor progression [54, 55]. Thus, during the earlier phase of tumor progression, the metabolic autonomy and invasive capacity of the tumor cells would induce their premature migration to the peripheral tissue [56].

Metabolically active cancer cells could induce interstitial edema [57]. There is increasing evidence that conditions characterized by an intense inflammatory response are associated with alterations in cellular membrane potential, with subsequent depolarization and abnormal ion transport. Moreover, disturbances in ion transport are associated with intracellular as well as interstitial edema [58]. Interstitial fluid flow is elevated in tumors, thus favoring the diffusion of solutes and proteins and inducing a substantial influence on cancer cells [59, 60]. The tumor interstitial fluid is absorbed by lymphatic capillaries and drains through lymph nodes in the thoracic duct, where it reaches the blood via the great veins of the neck [60]. Tumor interstitial fluid is suggested to be a rich sample for discovering biomarkers [61].

Compared with nonneoplastic tissue, the tumor stroma contains increased amounts of collagens, proteoglycans, and glycosaminoglycans [61, 62]. The accumulation of glycosaminoglycan fragments especially has been proposed as an important mechanism for edema formation because of its hydrophilic properties [63]. Glycosaminoglycans, that is, hyaluronan, are long unbranched polysaccharide chains which tend to adopt highly extended random-coil conformations and occupy a huge volume for their mass [63]. They attract and entrap water and ions, thereby forming hydrated gels, while permitting the flow of cellular nutrients [63]. Thus, interstitial edema could favor nutrition by diffusion through the malignant tumor. Under inflammatory conditions, hyaluronan is more polydisperse with a preponderance of lower-molecular forms and favors edematous infiltration of the tissues as well as the interstitial fluid flow and the tissue lymph pressure gradient [54, 63, 64].

**Figure 3:**
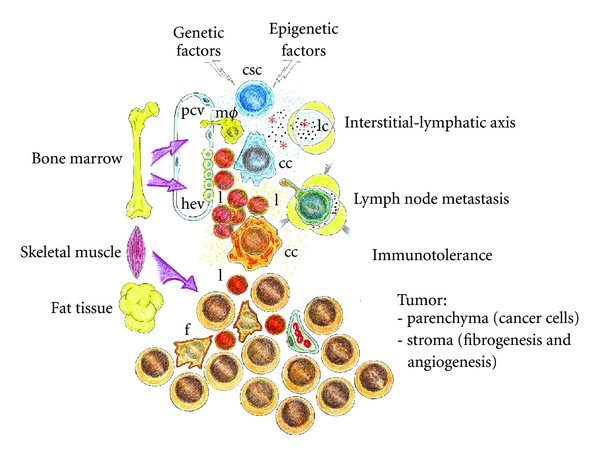
*Successive and overlapped stages of tumorigenesis.* Genetic and epigenetic factors stimulate the formation of a cancer stem cell that invades the interstitial space favored by the inflammatory interstitial-lymphatic axis, which stands out the tissue circulation of fluid and the cellular migration. The tumor cell, by means of using the natural and adaptive immune mechanisms, becomes immunotolerant, which favors the following phases of tumor development. Then, the cancer cell induces the creation of a stroma formed by a special type of granulation tissue, and this allows for the creation of a tumoral parenchyma provided with functional heterogeneity. Finally, this heterogeneous tumor mass plunders the trophic stores of the host inducing cachexia.

Tumor interstitial fluid represents the early microenvironment of the tumor cells [57, 59]. One may therefore envisage that access to tumor interstitial fluid bathing the cancer cells is considerably important in order to understand how tumors develop and progress [57, 59]. It could be suspected that during this early phase of the inflammatory cancer cell response, while edema progresses, the lymphatic circulation is simultaneously activated and this circulatory switch establishes an interstitial-lymphatic tumoral axis by which lymph can reach the systemic blood circulation [60]. In this way, lymphatic tumoral vessels are transformed in routes for trafficking through the body, exploited not only by immune cells but also by cancer cells [65]. Nevertheless, the interstitial tumoral fluid flow associated with edema can have important effects on tumoral tissue morphogenesis and function, cancer cell migration, and differentiation, and matrix remodeling, among other processes [66] (Figure 3). 

A number of studies in animal tumor models have established the concept that tumors, rather than just accidentally invading preexisting lymphatic vessels in their vicinity, can actively induce tumor-associated lymphangiogenesis by secreting appropriate growth factors, such as vascular endothelial growth factor (VEGF)-C, VEGF-D, or VEGF-A [65]. The formation of new lymphatic vessels, termed lymphangiogenesis, is often observed around or within the tumors [67]. With tumor progression, cancer cells secrete lymphangiogenic cytokines and growth factors which result in the formation of lymphatic vessels [68]. Tumor-associated lymphangiogenesis leads to a more extensive drainage network to capture the increased interstitial fluid flowing from the tumor cells and through the microenvironment [60]. Lymphatic vessels are also active modulators of immunity. The tumor associated with the lymphatic system may affect not only the local microenvironment, but also the host immune response against the tumor [69]. Recent findings raise the possibility that the tumor-associated lymphatic vessel and draining lymph nodes may be important in tumor immunity, which in turn govern metastasis. Peritumoral lymphangiogenesis associated with increased drainage to the lymph nodes could activate and maintain tumor tolerance by the host [69]. Hence, targeting lymphangiogenesis by developing antilymphangiogenesis agents might constitute a novel way to prevent lymphatic progression in some tumors [70].

It has been shown that tumoral lymphatic vessels could display a retrograde draining pattern. Today, the mechanisms of tumoral lymphatic dysfunction are not entirely known [68]. It is hypothesized that the rapid growth of tumor results in tissue edema, which generates mechanical forces to compress the lymphatics, or tumor cells can destroy the intratumoral lymphatic structure [68]. However, the high colloid osmotic pressure and protein concentration of the tumoral interstitial fluid [59] associated with the tumoral lymphatic dysfunction could favor cancer cell trophism by diffusion, modulate their metabolic microenvironment, and, therefore, play a key role in tumor growth and metastasis.

### 4.2. The Adoption of a Leukocytic Phenotype by Cancer Cells

The cancer cell could express a leukocytic phenotype with anaerobic glycolysis as the main source of energy, which permits lymphatic migration and invasion of the host [44–46].

Hypoxic tumor cells may suffer oxidative and nitrosative stress, like what occurs to leukocytes in the chronic inflammatory response by the generation of reactive oxygen and nitrogen species. Increased oxidative damage levels in malignant cells could result from more reactive species formation with unaltered antioxidant defenses, unaltered reactive species formation with decreases in antioxidant defenses, failure to repair oxidative damage, so that levels rise, or any combination of the above [71]. Overall, evidence supports the view that at least some malignant cells produce more reactive species for its own benefit, and the antioxidant defense and repair activities may sometimes rise, but not enough to cope with the extrareactive species [71].

In most inflammatory responses, the actions of reactive species are mediated by the I*κ*B kinase/nuclear factor (NF)-*κ*B (IKK/NF-*κ*B) system, and in turn, this system can be regulated by hypoxia and/or reoxygenation [52, 72]. More specifically, the expression of inducible genes leading to the synthesis of cytokines, chemokines, chemokine receptors, adhesion molecules, and autacoids relies on transcription factors, and among the primary transcription factors, NF-*κ*B plays a main role in the regulation of inflammatory mediators [73]. In addition, reactive oxygen and nitrogen species generated by both oncogene-expressing cells and inflammatory cells could cause oxidative damage to host DNA resulting in activation of oncogenes and/or inactivation of tumor suppressor genes and various epigenetic changes that favor tumor progression [72].

The activated IKK/NF-*κ*B pathway may play a tumor-promoting role by protecting tumor cells from death, that is, antiapoptotic, or enhancing their proliferation [72]. However, although much attention regarding NF-*κ*B has focused on inflammatory responses and tumor development, the current upsurge of interest in stem cell biology has led to studies of the function of NF-*κ*B in stem cells of the bone marrow. NF-*κ*B has been suggested to regulate secretion of growth factors and cytokines in adult and neonatal stem cells from the bone marrow environment [74].

Tumors in their development share an array of inflammatory activation mechanisms with leukocytes, including toll-like receptors (TLRs) [32, 75], prostaglandin and leukotrienes' metabolism [76], mitogen-activated protein kinases (MAPKs), that is, extracellular signal-regulated kinases (ERKs), c-Jun NH2-terminal kinases (JNKs) and p38 isoforms (p38s) [77], deregulation of phosphoinositide 3-kinase (PCI3K)/phosphatase and tensin homolog (PTEN)/Akt pathway [78], infiltrating tumor-associated macrophages (TAMs) [31] and lymphocytes T and B [34] and microRNA expression [36].

The activation of a leukocytic phenotype by the tumor cells could imply their transition to a more elaborated inflammatory response. Hence, tumor cells may usurp key mechanisms by which inflammation interfaces with cancer to further their colonization of the host [46].

The association between inflammation and cancer can be established by studying the influence of inflammatory cells in tumor progression, including neutrophils, eosinophils, macrophages, myeloid-derived suppressor cells, mast cells, and lymphocytes [1, 31, 35]. However, opposing effects of inflammation on cancer have been described, and, at the risk of oversimplifying for the sake of clarity, it can be said that acute inflammation counteracts while chronic inflammation promotes cancer development [79, 80]. Nevertheless, such a link may not be as simple as the one currently portrayed because certain types of inflammatory processes in skin, such as psoriasis or atopic dermatitis, and possibly other tissues as well, may also serve as a tumor suppressor function [81].

Chronic inflammation is involved in shaping the tumor microenvironment and has been referred to as “host reaction,” although it might be more appropriate to think of it as “tumor promoting” reaction [31]. In this way, cancer cells could reconvert the inflammatory host response to obtain an immunosuppressive inflammatory microenvironment and thus may escape from the host aggression [1, 31, 82]. An upregulation of immune tolerance by cancer cell could be induced using adaptive immunity [31, 34, 82, 83]. On the contrary, an inflammatory host response that switches the nature of the tumor promoting an immunosuppressive microenvironment could kill tumor cells and produce cancer regression [31, 82, 84].

Independently of the action of host leukocytes, and owing to the great plasticity of malignant cells, we could suspect that among the wide variety of phenotypes that they could express, it has been shown that tumor cells co-opt some of the signaling molecules of the host innate immune system, like chemokines, selectins, and their receptors, for invasion, migration, and metastasis [84]. Inflammation-associated products can also be secreted by the tumor cells themselves, giving rise to tumor microenvironment, which is overloaded and enriched with inflammatory factors with marked immunosuppressive abilities [80, 85]. In addition, immune cells in the tumor microenvironment not only fail to exercise antitumor effector functions, but also are co-opted to promote tumor growth and metastasis [31, 86] (Figure 3).

The hypothetical activation by chemokines of the leukocytic phenotype in the tumor cells could permit these cells to fulfill functions characteristic of activated inflammatory cells. For example, functions associated with neutrophils, such as the hyperproduction of extracellular proteases, including MMPs and other protease enzymes that carry out a true extracellular digestion of the basement membrane and the extracellular matrix, also aid invasiveness in the early stages of the disease [55, 87]. The extracellular matrix is a reservoir for many molecules, including growth factors, and cytokines, which are only released upon their dissolution [88]. Also, cancer cells can present pseudopodia formation and directional migration [89]. Other functions seem to correspond to a monocyte-macrophage phenotype in the sense that tumor cells migrate to the regional lymph nodes through the lymphatic capillaries [69, 90]. Moreover, the so-called fusion theory explains that the acquisition of the metastatic phenotype occurs when a healthy migratory leukocyte fuses with a primary tumor cell. And interestingly enough, the fusion of genetic and cytoplasmic material between cells of different origins is an important physiological process during development [91].

Cancer cells dissociated from the primary tumor could change their phenotype to become more autonomous, express specific adhesion molecules, produce lymphangiogenic factors and eventually evade the host defense [68]. The hypothetical adoption of a leukocytic phenotype by most tumor cells would also imply the acquisition of a similar metabolism. In this situation, like the activated leukocytes, cancer cells function would require glycolysis as the main source to obtain substrates and energy [92]. In this sense, the metabolic regulation of cancer metastatic cells could be closely related to a specific mutational activation of oncogenes and inactivation of tumor suppressor genes [93]. In contrast to normal cells, cancer cells tend to convert glucose into lactate even in the presence of sufficient oxygen, a term called “the Warburg effect” or aerobic glycolysis [94]. Glutamine is also a major cancer cell energy and anabolic substrate [93]. The oncogenes, Akt, Myc, and Ras, MAP-kinase, PI3K, and mammalian target of rapamycin (mTOR) pathways, and HIF can stimulate the transcription of a number of genes that encode the proteins that mediate the glycolysis and glutaminolysis pathways [93, 94]. This metabolic characteristic of the tumor cell in this stage would explain why tumors grow in the lymph node, regardless of their angiogenic ability [95]. In turn, nutrition of cancer cells during the expression of a leukocytic phenotype could also be based on extracellular, that is, proteolytic enzymes, and intracellular, that is, phagocytic, functions including autophagy and digestion [44, 45].

During the adoption of a leukocytic phenotype, soluble factors could push tumor cells towards premature migration to peripheral tissue [45]. Considerable progress has been made in recent years regarding how tumor cells circulating in the blood and lymphatic system interact with and extravasate into secondary sites and what determines whether these disseminated tumor cells survive, remain dormant, or go on to form macrometastases [96]. Hence, the leukocytic phenotype would favor the homing of metastatic tumor cells to specific organs, especially those where populations of resident macrophages are abundant, that is, lung (alveolar macrophages), liver (Kupffer cells), brain (glia), and bone (osteoclasts) [44]. Particularly, specific chemokines in distant organs and chemokine receptors on the tumor cells dictate a metastasis pattern according to the cancer cells [97].

Tumor-cell-derived factors, which could also be partially contributed by tumor-infiltrating macrophages [31], have been found to induce the expansion of the lymphatic network in the lymph nodes, even before the arrival of the metastatic cancer cells, as if to create a favorable environment for the future metastasis [98, 99]. Also, disseminated tumor cells in bone marrow can be detected in 20–40% of cancer patients without any clinical or histopathological signs of metastasis [100]. The particular bone marrow environment may induce these cells to survive and disseminate later into other distant organs. This “dormant stage” of disseminated tumor cells may explain why these cells are relatively resistant to chemotherapy [101].

The adoption of a leukocytic phenotype by cancerous cells could be associated with an increased degree of lymphangiogenesis. In animal models, a strong relationship between lymphangiogenesis and survival has been shown [67]. Lymphangiogenesis at the site of the primary tumor as well as in the draining lymph node actively contributes to metastatic cancer spread, and its inhibition might be of interest for preventing tumor metastasis [67, 98, 102]. Lymphangiogenesis not only is crucial for cancer cells to metastasize, but also offers the tumor the possibility to disseminate inflammatory mediators in the host, which would produce a systemic inflammatory response syndrome (SIRS) [45, 103]. The SIRS, mediated in part by proinflammatory mediators including cytokines, plays a role in the genesis of cachexia associated with both critical illness and chronic inflammatory diseases [104]. The systemic inflammatory and immune response to the presence of a tumor is manifested in different ways, according to the type and extent of the tumor. Fever, night sweats, weight loss, fatigue and malnutrition may all be attributable to tumor-induced inflammation [3]. Cancer cachexia is a continuum with three stages of clinical relevance: precachexia, cachexia, and refractory cachexia, but not all patients traverse the entire spectrum [105]. Additionally, although an acute phase protein response is usually produced, the C-reactive protein in serum is the most widely accepted index of systemic inflammation, and cachexia can be present in the absence of overt systemic inflammation [105]. Thus, the host, as a yolk-sac-like structure, becomes a vitellum supplier for the tumor growth. 

The tumor cell, through the lymphatic system, can also modulate the host immunity, using its own antigen production. Tumoral antigens in the interstitial fluid are collected into open-ended lymphatic capillaries, which can form a mesh-like network by lymphangiogenesis, which could then be transported to the lymph nodes [106]. However, tumoral antigens in prenodal lymph, before entering the node and once inside, could be filtered by dendritic cells and nodal antigen-presenting cells, respectively [106]. Thus, lymph-carried tumoral antigens might have particular relevance for the induction and maintenance of peripheral tolerance to cancer cells.

The functional plasticity of dendritic cells allows for adapting the immune system to mount functionally distinct types of responses, including the immunologic tolerance that could contribute to tumor development [82]. In this way, it is accepted that dendritic cell vaccines will be offered to patients either with early- or late-stage disease to elicit a strong and long-lived antigen-specific T-cell immunity [82].

Additionally, lymphatic vessel density can increase in the chronic inflamed tumor tissue, although without reestablishing an efficient lymphatic connection with the draining lymph nodes [107]. This defective lymphatic drainage, associated with the enduring local tumoral antigen stimulation, could be a crucial trigger of the cascade of events leading to lymphoid neogenesis [108]. De novo lymphoid tissue formation or tertiary lymphoid organs occur during states of chronic inflammation [69]. Tertiary lymphoid organs may accumulate tumor antigens and tumor antigen-presenting cells, bypassing the lymph node function and therefore circumventing their tolerance-maintaining function [69, 108]. In addition, myeloid-derived suppressor cells, a heterogenic population of immature myeloid cells that consists of myeloid progenitors and precursors of macrophages, granulocytes, and dendritic cells, use a number of mechanisms to suppress various T-cell functions as well as to induct regulatory T-cells [109].

### 4.3. Granulation-Tissue-Related Cancer Cells

Cancer cells, to grow inside the host, need to induce the formation of a stroma to change in an organ. Therefore, the comparison of tumors with wounds that do not heal includes, among other characteristics, the development of a stroma in which the formation of fibroblasts, through a process named fibroplasia, mainly takes place [6]. This phase of skin wound healing, also called granulation phase, is basically characterized by active fibroplasia and angiogenesis [110] (Figure 3).

The role of fibroblasts in wound healing has been extensively studied over the past years. Fibroblasts and myofibroblasts mainly produce the new extracellular matrix necessary for supporting cells and blood vessels, which provides nutrients and oxygen needed for cell growth and proliferation [110]. This new matrix consists of collagen, proteoglycans, and fibronectins produced by the fibroblasts. Fibroblast activity is predominantly regulated by platelet-derived growth factor (PDGF) and transforming growth factor *β* (TGF*β*) [111]. Growth factors involved in fibrogenesis also include fibroblast growth factor-2 (FGF-2) and hepatocyte growth factor (HGF), which are also chemotactic factors for mesenchymal stem cells. Complementary to this, it has been demonstrated that keloid-derived fibroblasts induce higher mesenchymal stem cell migration toward themselves than normal fibroblasts [112]. Also, it must be noted that keloids are locally aggressive scars that typically invade healthy surrounding tissues [112]. Recent studies indicate that mesenchymal stem cells from the bone marrow provide “fibroblasts” to the skin in adults and are thought to enhance skin repair/regeneration [33, 113]. 

In recent years, the tumor stroma has become the focus of intense research. In particular, fibroblasts, a heterogeneous collection of mesenchymal cells, are among the most abundant cell types in the microenvironment of solid tumors [37]. Cancer-associated fibroblasts, through the release of cytokines and growth factors, could modulate the cancer stem cell phenotype and could also lead to enhanced angiogenesis [33, 37]. It has been suggested that cancer-associated fibroblasts may be derived from tumor cells that undergo epithelial-mesenchymal transition [114]. One characteristic of the cancer-associated fibroblasts is their heterogeneity, which is also expressed through the formation in solid tumors of a very different profile of extracellular matrix proteins [37]. Mesenchymal stromal cells also can suppress immune responses, favoring tumor cells' escape from the host immune response [115].

Angiogenesis characterizes a phase of cancer evolution that permits numerous substances, including hormones, to be transported by the blood. Angiogenesis requires migration of endothelial cells into the interstitial space with the subsequent proliferation and differentiation into capillaries [44]. Tumors induce angiogenesis by activating tumor stromal cells. The release of angiogenic factors from the extracellular matrix through new formed epitopes promotes angiogenesis. Thus, the neoplastic cells switch to an angiogenic phenotype [116]. However, tumor angiogenesis produces a tumor-associated vasculature that is chaotic, both in structure and function. Although angiogenesis supplies a growing tumor with nutrients and oxygen, the neo-vasculature is poorly formed often with leaky blood vessels that do not link the arterial to the venous circulation but is rather dead-end [42, 117]. This characteristic impairs tumor blood flow and the delivering of oxygen [118], but they favor its growth since without angiogenesis tumors rarely grow to larger than 2 to 3 mm [119]. However, the tumor during its growth seems to prioritize the venous-lymphatic circulation in detriment of the arteriovenous circulation, which is characteristic of the specialized tissues. This circulatory switch perhaps could offer some metabolic advantages to the tumor biology; for instance, it would favor an environment poor in oxygen, strengthening therefore an efficient anaerobic metabolism that forms biomass, and to the blood endothelium with an inflammatory phenotype; it would give more permeability to molecules and cells and it would also enrich the composition of the interstitial space. In essence, a “reactive stroma” would be created [59, 120]. The normal stroma in most organs contains a minimal number of fibroblasts, whereas a reactive tumor stroma could be associated with an increased number of cells, including fibroblasts, enhanced vessel density, and protein deposition [59].

Cancer cells are also associated with high endothelial venule development. Lymphocytes get into lymph nodes from the blood through high endothelial venules. T and B cells subsequently move into the T zone and B-cell follicles, respectively, and then migrate in a stromal-guided random walk [121]. In contrast to the flat endothelial cells that line other types of blood vessels, high endothelial venule endothelial cells are almost cuboidal, and they selectively express certain tissue-specific adhesion molecules and chemokines [122]. High endothelial venules are mainly found in the paracortical and interfollicular areas of the lymph node and are surrounded by an intricate stromal network consisting of fibroblastic reticular cells. However, high-endothelial venule-like structures are also associated with tertiary lymphoid organs, which represent highly organized lymphoid tissues induced by inflammation [122]. In addition, recruitment of lymphocytes and dendritic cells to inflamed lymph nodes by trans-high-endothelial venule migration involves the mediation of heparan sulfate [123].

Tumors build their blood vessels by mechanisms that involve endothelial cell response, including circulating endothelial precursors and circulating endothelial cells, to tumor signaling [97]. Recent works provide evidence indicating that the state of endothelial cell activation in the tumoral niche, rather than angiogenesis itself, may dictate tumor dormancy or escape. In essence, the crosstalk between tumor cells and stromal cells, including endothelial cells within the tumor microenvironment, mediates tumor evolution [97, 124].

Within the factors that play a role in initiating tumoral neovascularization, the “classical,” that is, VEGF, fibroblast growth factor-2 (FGF-2), also termed basic fibroblast growth factor (bFGF), platelet-derived growth factor (PDGF), transforming growth factors (TGFs), and angiopoietins (Angs), and the “nonclassical” mediators of angiogenesis, that is, erythropoietin, angiotensin II, endothelins, and thyroid hormones, stand out [125].

However, as it has been previously explained, tumor vasculature is typically aberrant [117] with a reduced pericyte coverage, which in turn destabilizes vascular integrity and function [126]. Additionally, vascular heterogeneity could be associated with subpopulations of cancer cells that differ in their energy-generating pathways, macromolecular biosynthesis, and redox control [42, 43, 126, 127]. These populations may function symbiotically since the cells proximal to a better-oxygenated tumoral tissue consume oxygen with glucose and glutamine, serving as substrates and secreting lactate that can be used by another subpopulation of hypoxic cancer cells as their main energy source [42, 126]. In a similar compensatory way, the activation of HIF in the hypoxic cancer cell subpopulation or in stromal cells within the tumor could augment their vascularization and oxygenation. This change could be associated in turn with tumoral blood flow redistribution, rendering other areas of the tumor ischemic, and consequently with a hypoxic metabolism that induces an increase in tumor invasiveness and risk of metastasis [50]. Thus, it can be suspected that the metabolic and functional heterogeneity of the tumors, which is imposed by their stroma, that is, granulation tissue-like, is dynamic. This tumoral dynamic heterogeneity could constitute a favoring factor of chronicity.

## 5. Inflammatory Cancer Phenotypes and Related Metabolisms: Regarding the Need for a Metabolic Staging of Cancer

Given the hypothetical plasticity of malignant tumor cells, the above-mentioned inflammatory phenotypes would need the corresponding metabolic plasticity. Hence, the mechanisms that govern tumoral evolution could be based on the increasing metabolic capacity of the tumor cell to use oxygen over the inflammatory phenotypes that supposedly drive the successive phases of release, migration, and proliferation [44–46]. Since it has been proposed that these phases of tumoral evolution go from hypoxia to the progressive development of an oxidative metabolism, it has been speculated on whether the tumor cell reproduces most of the successive stages by which life passes [44] from its origin without oxygen until it develops an effective, although costly, system for the use of oxygen [128]. If so, in the successive metabolic switches that cancer undergoes, it acquires an increasing ability to both invade the host and use its sources of substrates until its metabolic reserves are all used up [44, 45].

The ability of cancer cells to change their metabolism and, therefore, their inflammatory phenotypes is one of the fundamental reasons why metabonomics offers a platform for biomarker development in the field of oncology [129]. Yet cancer metabolism represents an ideal field for metabolic profiling because of the way its metabolism differs substantially from normal cells, especially that of the glucose and phospholipid [130]. Tumoral metabolic profiling, not only could allow us to study each tumoral inflammatory phenotype but would also offer the chance of correlating tumoral metabolism with its invasiveness to the host.

The hypoxic phenotype would characterize the initial step of tumoral evolution. In this early phase of the cancer cell response, it could be considered that hypometabolism, anaerobic glycolysis with lactate production, and a low energy expenditure [131] could be related to a primitive cellular trophic mechanism, like diffusion. [44, 45]. In this supposed state of cancer cell stunning, complex or specialized functions could not be expressed. It is conceivable that near-anoxic cancer cells initiate a transcriptional response that compensates the low metabolic demand with reduced oxygen availability. The key player of this adaptive response is HIF [132–134]. However, the HIF complex modulates signaling by Notch, a critical regulator of undifferentiated stem and progenitor cells [134].

The Notch signal pathway is involved in cell fate decisions during normal development but also in the genesis of several cancers [135, 136]. Activation of Notch leads to proteolytic cleavage of the intracellular domain of Notch. Thus, translocation of Notch intracellular domain into the nucleus induces the transcriptional activation of Notch target genes [135, 137]. Notch target genes include proteins and factors involved in the control of the cell cycle and survival processes [137, 138]. In human cancers, activation of Notch signaling also can establish crosstalks with many oncogenic signaling pathways, such as developmental signals, for example, Wnt and Hedgehog signaling [138]. In particular Hedgehog plays a key role in a variety of processes such as inflammation, carcinogenesis, and embryogenesis [137, 138].The signaling pathways that control self-renewal of stem cells are an essential element for tumor survival. That is why cancer stem cells use many of the above-mentioned signaling pathways that are found in normal stem cells, such as Wnt, Notch, and Hedgehog [137, 139].

Tumor metabolism related to the leukocytic phenotype could be able to manage the increased oxidative and nitrosative stress and hypermetabolism imposed by reoxygenation. Recently, the presence and generation of reactive oxygen species attracted increased interest as a microenvironmental factor as part of reoxygenation affecting the survival of tumor cells [140]. In particular, upon reoxygenation, hypoxic cells could experience apoptosis, but there is also evidence that malignant cells can increase their tolerance in response to adverse metabolic conditions [127, 140]. In turn, metabolic stress situations, such as oxidative stress, low pH, or low glucose, are likely to be major determinants of the metabolic phenotype. The regulation of this “metabolic flexibility” is poorly understood and will require a much greater degree of understanding if effective therapeutic strategies targeting metabolism are to be developed and effectively deployed [127].

The pentose phosphate cycle is composed of two branches: the irreversible oxidative pentose pathway that converts glucose-6-phosphate to ribose phosphates thereby yielding two moles, NADPH H+, per mole glucose, and the nonoxidative pentose phosphate pathway that reversibly converts three pentose phosphates into two hexose phosphates, for example, fructose-6-phosphate, and one triosephosphate [131, 141]. The normal cells produce most of the ribose-5-phosphate for nucleotide synthesis through the oxidative pentose pathway; however, in tumor cells, the nonoxidative pentose phosphate pathway is the main source for ribose-5-phosphate synthesis. In turn, it is accepted that there are major differences in the relative share of these two pathways in the delivery of pentose phosphates when comparing slow and fast-growing carcinoma [131].

In particular, in response to oxidative-stress, central carbohydrate metabolism could be reconfigured, so that the metabolic flux reroutes from glycolysis into the pentose phosphate pathway, which allows cells to mount an effective response to this cellular stress [142, 143]. Activation of the pentose pathway also could contribute to many of the unique metabolic requirements of tumor cells. Through its generation of NADPH, the oxidative arm of the pentose phosphate pathway provides reducing power to drive anabolic metabolism [143]. If so, deficiency of the pentose phosphate pathway could influence the development of a diverse variety of oxidative stress-associated human diseases, ranging from autoimmune diseases to carcinogenesis [144].

The mammalian target of rapamycin (mTOR) is an evolutionary conserved Ser/Thr kinase, which could play a major role in the metabolic reprogramming of tumor cells [143, 145]. In addition to its well-known roles in promoting protein synthesis and inhibiting autophagy [43], mTORC1 has been found to stimulate glucose uptake, its conversion to glucose 6-phosphate, and metabolic flux through both glycolysis and the oxidative arm of the pentose phosphate pathway [143]. As mTORC1 signaling is aberrantly elevated in the majority of genetic tumor syndromes and sporadic cancers, this pathway is poised to be a major driver of this metabolic conversion of tumor cells [146].

Notch-mediated signals can upregulate several factors that in turn transmit bidirectional signals among cancer cells expressing both ligands and receptors. Therefore, it is not surprising that Notch signal crosstalks with many oncogenic signaling pathways, such as developmental signals including Wnt and Hegdehog, as well as transcriptional factors, for example, NF-*κ*B [138]. In particular, IL-1 activates Notch signaling pathways probably through NF-*κ*B pathway. Notch pathway is also a critical downstream target of IL-6 [138]. It has been accepted that the family of NF-*κ*B transcription factors is involved in the expression of genes related to innate and adaptive immunity, which suggests that this signaling pathway could also favor the expression of the leukocytic phenotype in cancer cells. Therefore, Notch signaling could also be required to convert the hypoxic stimulus into epithelial-mesenchymal transition favoring the motility and invasiveness of cancer cells [140, 147].

Finally, sonic hedgehog (Shh), a member of the Hedgehog family, is involved in numerous aspects of embryonic development including angiogenesis and lymphangiogenesis [148]. Shh is an established morphogen critical to the development of the vascular system, but in adult ischemic pathologies, it stimulates the production of angiogenic factors, including VEGF-A and angiopoietin-1. It also promotes endothelial cell chemotaxis [148, 149]. In the ischemic cancer cell, Shh acting concomitantly with the Notch signaling pathway could also stimulate the production of angiogenic factors [135, 137, 149]. These newly characterized pathways have been functionally implicated in the development and tumor-associated angiogenesis. They also illustrate the complex regulation of endothelial cell phenotypes [126]. As a result, a broader concept of the tumoral angiogenic process is needed for its better study and for a better comprehension of the involved mechanisms since both blood and lymphatic vessels are major vascular components of the tumor. This is the reason why the tumor-associated blood/lymph angiogenesis is accepted as a process induced by complicated cytokine networks, mediated by the paracrine and autocrine interactions between tumor cells and stromal cells [150].

 Thus, tumor nutrition mediated by blood and lymphatic capillaries could be established thanks to angiogenesis. The new functional properties of tumor microcirculation could include the exchange of oxygen, nutrients, and waste products [44, 45] and favor tumor growth, invasion, and metastasis [151, 152]. Recent information on mitochondrial metabolism in malignant neoplasia emphasizes that, although tumor cells maintain a high glycolytic rate, the principal ATP production may be derived from active oxidative phosphorylation [153]. The result of these metabolic characteristics is that tumors burn glucose while consuming muscle protein and lipid stores of the organism. As a result, tumor metabolism gives them a selective advantage over normal cells [154].

## 6. Cancer Cell Meets Amniotic and Vitelline Functional Axes

The concept that cancer and embryonic cells have much in common is an old idea. The morphological resemblance between cancer cells and the cells of fetal tissues has been repeatedly discussed in current biomedical literature [155] (Table 3). In the mid-19th century, upon observing cancer tissue under the microscope, the forefathers in pathology noticed the similarities between embryonic tissue and cancer and suggested that tumors arise from embryo-like cells [156]. More recently, it became clear that neoplastic cells possess a more embryonic phenotype than their tissue of origin and that this involves the expression (or reexpression) of embryonic genes [157]. Both embryos and tumors display similar antigens, elaborate angiogenic growth factors, and subvert apoptotic cell death. Furthermore, they may both escape immune destruction by similar mechanisms [157]. 

Recent studies have shown that it is possible to reprogram the melanoma tumorigenic phenotype by exposing melanoma cells to factors present in the embryonic microenvironment [158]. This suggests that melanoma cells may share some characteristics with stem cells that allow them to respond to the ones from the embryonic microenvironment [159]. It became clear that neoplastic cells posses a more embryonic phenotype than their tissue of origin and that this involves the re-expression of embryonic genes [157, 159]. The convergence of embryonic and tumorigenic mechanisms has indeed allowed suggesting the use of embryonic vaccines against cancer in the past [157].

Tissue interstitium under conditions of long-lasting inflammation is associated with oxidative stress, edema, enzymatic stress, persistent leukocyte stimulation, lymphangiogenesis, angiogenesis, and fibrosis [160, 161]. It has been proposed that if the insult is sustained, for example, chronic inflammatory response, additional proinflammatory mediators can activate a wide variety of leukocytes including macrophages and lymphocytes, which can contributes to further tissue destruction and inflammation [162]. However, prolonged inflammation in wounds contribute to the development of fibroproliferatiove scarring, in other words, keloids and hypertrophic scars [163]. Moreover, in autoimmune diseases, persistent antigenic stimulation recruits endogenous mesenchymal stem cells to the site of the lesion that contributes to the fibrotic evolution [164].

Transforming growth factor (TGF)-*β* signaling in stromal cells, for example, fibroblasts, also exerts significant effects on tumor development and growth. It has been shown that TGF-*β*, an important tumor suppressor, also regulates infiltration of immune cells as well as fibroblasts in the tumor microenvironment and promotes tumor progression [165]. Fibroblasts could also produce c-Kit ligand, the most important mast cell growth factor, while mast cells' proteases released from activated mast cells have an important effect on fibroblasts [166]. There is evidence that cancer-associated fibroblasts have a cancer-promoting phenotype [167]. Consequently, cancer-associated fibroblasts could produce significant extracellular matrix remodeling during tumor progression mediated by tumor-specific extracellular matrix proteins and matrix metalloproteinases isoforms [37].

Adult mesenchymal stem cells can be defined as multipotent cells able to differentiate into various types including specialized mesenchymal cells. The behavior of mesenchymal stem cells towards the immune system is context sensitive. Although the antifibrotic effects of mesenchymal stem cells have been demonstrated, the molecular mechanisms behind this effect are not yet fully understood. However, the angiogenic support provided by mesenchymal stem cells is considered the more supportive effect, because reestablishment of blood supply is fundamental for the recovery of damaged patients [168].

In contrast to adult wound healing, the early gestation fetus has the remarkable ability to heal skin wounds without scarring [169, 170]. There are numerous intrinsic and outrinsic differences between the fetus and adult that may influence wound healing. The fetal wound is continuously bathed in amniotic fluid rich in growth factors and extracellular matrix components such as hyaluronic acid, type III collagen, and matrix metalloproteinases. In addition, fetal wound healing is characterized by a rapid upregulation in progenitor cells of genes involved in cell growth and proliferation, with decreased platelet aggregation and degranulation, compared to adult wound healing [170].

One intrinsic difference also includes fetal tissue oxygenation. The fetus has a very low pO2 since there is a large transplacental oxygen gradient between maternal arterial and umbilical venous blood. Consequently, the fetus can heal in a relatively hypoxemic environment [169].

Epithelial to mesenchymal transitions (EMTs) are transdifferentiation programs that are also required for tissue morphogenesis both during embryonic and cancer development [171, 172]. The conversion of epithelial cells to mesenchymal cells is fundamental for embryonic development and involves profound phenotypic changes, including the loss of cell-cell adhesion and the acquisition of migratory and invasive properties [114, 171]. Recent evidence suggests that normal stem cells and cancer stem cells share a mesenchymal phenotype that enhances their ability to preserve stemness, to retain migratory properties, and to respond to different stimuli during expansion and differentiation [171]. Thus, EMT induction in cancer cells results in the acquisition of invasive and metastatic properties [171, 172]. Interestingly enough, these invasive cells, with both a stem-cell-like and mesenchymal phenotype, can generate an epithelial-like structure by mesenchymal to epithelial transition (MET) and, therefore, could be involved in the formation of macrometastasis [171]. Furthermore, it is accepted today that metastasis progression should be considered an independent and parallel process in tumorigenesis governed by the EMT that occurs among tumor cells [91, 114, 172]. In addition, although the majority of tumors are epithelial, they also exert mesenchymal characteristics [91, 171, 172].

An EMT process also occurs in mammalian embryos during gastrulation [114]. Gastrulation is a developmental phase that delineates the three embryogenic germ layers, named ectoderm, endoderm, and mesoderm. Haeckel coined the term gastrulation derived from the Greek word *“gaste,”* meaning stomach or gut, that transforms the rather unstructured early embryo into a gastrula with several specific characteristics: the three primary germ layers are formed; the basic body plan is established, including the construction of the rudimentary body axes; the cells assume new positions, allowing them to interact with cells that were initially not close to them [173]. The nascent mesoderm generated during gastrulation could involve an internalization process of extraembryonic phenotypes [114]. In essence, gastrulation could be represented as the creation of an interstitial space in which extraembryonic, that is, amniotic and vitelline, functions are expressed using mesenchymal cells. If so, the mesoderm would represent the vehicle or the mediator for the internalization of the extraembryonic functions into the embryos [8] (Figure 1).

 The mesenchymal state is associated with the capacity of cells to migrate to distant organs and maintain stemness allowing their subsequent differentiation into multiple cell types during development and the initiation of metastasis [114, 171]. In amniotes, members of the TGF-*β* superfamily induce gastrulation and Nodal signaling together with fibroblast growth factor (FGF) and control the specification of the mesendoderm in all vertebrates [171]. Mesenchymal stem cells are a heterogeneous population with several subgroups of cells with different proliferative and differentiation potentials [9]. Mesenchymal stem cells support hematopoiesis and are able to differentiate towards the mesodermal lineage to generate smooth muscle cells, fibroblasts, pericytes, myofibroblasts, osteoblasts, chondrocytes, and adipocytes [9].

Hypoxia is commonly associated with conditions such as tissue ischemia, inflammation, and solid tumors. However, hypoxic niches in the developing embryo are associated with regulation of cellular differentiation [174]. In this sense, mammalian development occurs in a relatively oxygen-poor environment and before the circulatory system is established. Therefore, it would seem logical that blood vessel patterning could be fine-tuned by local hypoxic microenvironments that are encountered during embryogenesis, organogenesis, and tumorigenesis [174]. This is why it could be considered that hypoxia could influence the behavior of cancer stem cells and their progeny promoting a defective tumor angiogenesis with vascular-like networks [159].

Fibroblasts are among the most abundant cell types in the microenvironment of solid tumors. Carcinoma-associated fibroblasts promote tumor growth and invasion and stimulate angiogenesis [37]. However, the altered phenotype of carcinoma-associated fibroblasts with the production of an impaired extracellular matrix and favoring the interstitial infiltration by inflammatory cells [37] could be key factors for inhibiting the differentiation of the tumoral microcirculation. In turn, this immature microcirculation would be responsible for the tumor-uncoupled metabolic functions regarding the normal microcirculation, as well as its proliferative ability and invasiveness of the host.

The vast arrangement of the mesenchyma around and between the developing amniotic and yolk sac cavities suggests an important role of the mesenchyma in orchestrating embryo development. Mesenchyma isolated specifically from the amniotic membrane could differentiate into neuronal-like cells which are identified to secrete dopamine [175]. Cells derived from amniotic fluid also have a neuronal, dopaminergic phenotype [176]. These results allow for considering the amnion as an embryonic functional axis with strong neural potential [175, 176]. In addition, experimental and clinical studies have demonstrated that amniotic membrane transplantation has important biological properties, including anti-inflammatory, antimicrobial, antifibrosis, and antiscarring, as well as low immunogenicity [177, 178]. Amnion-derived multipotent progenitor cells secrete a unique combination of cytokines and growth factors, known as “amnion-derived cellular cytokine solution,” which establish a communication network between mesenchymal and epithelial cells during embryo development. That is why using the amnion to accelerate wound healing through its functions has been proposed, which regulates migration, proliferation, and differentiation of fibroblasts as well as of keratinocytes [179].

In turn, the extraembryonic visceral yolk sac in mammals is composed of two layers, that is, the visceral endoderm, which is active in endocytosis/digestion and has large lysosomes, and the underlying mesoderm layer [180]. In the embryonic mesoderm layer, “blood islands” develop supporting hematopoiesis and angiogenesis [181]. While formation of the various primitive hematopoietic populations is restricted to the yolk sac, progenitors of definitive hematopoietic cells that arise in the yolk sac may contribute to hematopoiesis in the embryo proper [181]. Also, a major function of the yolk sac is associated with the accumulation of carbohydrates, proteins, and lipids for embryo nutrition (*vitellum*) [16]. Particularly, the yolk sac plays a vital role in providing lipids and lipid-soluble nutrients to embryos during the early phases of development [16]. Interstitial lipid accumulation of cholesterol, a precursor molecule of many hormones, like aldosterone, corticoids, androgens, strogens, and progesterone, may favor fluid infiltration and cell migration, proliferation, and differentiation during embryo development [19].

Dissecting out the possible contribution of extraembryonic lineages to the embryo proper has been difficult, in large part because of the inaccessibility of the mammalian embryo within the uterus [181]. In addition, the term “extraembryonic” is somewhat a misnomer, as there is no clear anatomical or molecular demarcation to separate the embryonic from the extraembryonic tissues during the early steps of the development. Also, at later stages both are integral components of the developing embryo [182]. However, recent studies suggest that extraembryonically derived functions and cells make an increasingly significant and possibly exclusive contribution to the embryonic development [44, 181]. Thus, the molecular and cellular contribution made by both extraembryonic structures, that is, the amnion and the yolk sac, to the interstitial space located between them, namely, the mesoderm, are essential for organogenesis. Particularly, both in the amnion axis and in the yolk sac axis, the extraembryonic mesenchyma plays an important role [9].

The internalization of extraembryonic functions, that is, amniotic-like and vitelline-like, by mesenchymal cells not only could be a key process of the embryonic development, but also could be used by the postnatal organism when it suffers an injury and, therefore, needs to be repaired. Thus, an acute or chronic injury could induce a dedifferentiation process with the expression of different and overlapping inflammatory phenotypes that resemble similar phenotypes expressed during embryo development. Particularly, molecular and cellular amniotic and vitelline mechanisms involved in gastrulation would return [8].

Moreover, the relationship between inflammation and cancer also could be based on the orchestration of extraembryonic functions. If so, tumorigenic cancer cells could successively induce the expression of overlapping amniotic and vitelline-like phenotypes that promote the invasion, control, and remodeling of the interstitium. The amniotic-like phenotype could offer the cancer cells an interstitial-lymphatic axis [59], favoring transport, nutrition by diffusion, excretion, and bacteriostatic and anti-inflammatory protection [179]. In turn, the vitelline phenotype could favor the regulation of lipid metabolism genes, including cholesterol and eicosanoid homeostasis [76], hematopoietic/bone marrow control [113], and the induction of an “angiogenic switch” [117, 126] to permit tumor and metastatic growth. The integration of both extraembryonic phenotypes by the cancer cell would support the functional and metabolic heterogeneity needed to successively modulate their microenvironment during their development in the host (Table 2). 

The new point of view proposed in the current review would be based on considering the extraembryonic mechanisms as the expression of an ancient type of inflammatory response. Ancestral mechanisms of natural and acquired immunity immersed into the amniotic and vitelline axes would allow the acceptation of new and foreign tissues by the host. Therefore, the neoformed tissues, either physiological, that is, embryo and repair tissue, or pathological, that is, cancer, both of them being alien to the host organism, would need to use similar extraembryonic mechanisms to be grafted successfully.

The involvement of these extraembryonic mechanisms, which are individualized in each patient in tumorigenesis, would be a key inducing factor of the vast heterogeneity of cancer [183, 184]. Moreover, cancer cells could be modified through epigenetic modifications that alter gene-expression patterns. Like all the cells that constitute the human body, a cancer cell is a direct descendent of the fertilized egg from which the cancer patient developed. Compared with the fertilized egg, the cancer genome will also have acquired epigenetic changes with an altered chromatin structure and gene expression [185].

## 7. Conclusion

It could be concluded that cancer cells acquire the ability to invade the host organism through the recapitulation of extraembryonic, amniotic, and vitelline functions. In this way, through the hypothesized overlapping expression of these two extraembryonic functional axes, cancer cells would adopt the different phenotypes that they need to develop. Maybe this is the reason why pluripotent cancer-initiating cells would acquire the nature of embryonic cells. Thus, the cancer-initiating cells would have the amazing ability of inducing, for their own benefit, the expression in the host of functional extraembryonic mechanisms. 

The immunological properties of the extraembryonic functions would have the aim of inducing the acceptance of the embryo by the maternal immune system. In addition, the adoption during the postnatal life of the above-mentioned extraembryonic functions for developing new tissues during wound repair and tumorigenesis could have the same objective. Therefore, an appropriate immunological and trophic microenvironment would be created for coexisting with the host.

Since the tumor tissue is what induces the expression of extraembryonic functions in the host that favor its development, it would become an autonomous organism that progressively takes over its nutritional stores. This behavior could be compared with how the cuckoo adapts for reproduction. This bird uses foreign nests to lay its eggs, which will be incubated by other birds. After hitching, the newborn cuckoos throw out all potential competitors from the nest and thus monopolize the nutritional support of the deceived parents. In the same way, tumor cells act selfishly, compared to the altruistic behavior characteristic of the normal cells of the host. Hence, in cancer, new immunotolerant tissues monopolize the nutritional resources of the host. This behavior could represent a last attempt to survive this indomitable eukaryotic cell even at the expense of “murdering” its supporter.

## Figures and Tables

**Table 1:** Phenotypes expressed in the acute inflammatory wound healing response*. *

(i) *Ischemia-reperfusion phenotype *
(a) Oxidative and nitrosative stress
(b) Interstitial hydroelectrolytic alterations
(c) Increased lymphatic circulation (circulatory switch)

(ii) *Leukocytic phenotype *
(a) Infiltration by granulocytes and agranulocytes
(macrophages-lymphocytes)
(b) Lymphangiogenesis
(c) Lymph node reaction
(d) Wound immunotolerance
(e) Resolution

(iii) *Angiogenic phenotype *
(a) Endothelial cell proliferation
(b) Fibrogenesis
(c) New vascular network
(d) Epithelial regeneration

**Table 2:** Hypothesized cancer cell extraembryonic (amniotic and vitelline) and embryonic (gastrulation) phenotypes.

(i) *Amniotic-like phenotype *
(a) Abnormal ion transport
(b) Extracellular matrix permeability
(c) Diffusion
(d) Increased interstitial fluid (cytokines-substrates)
(e) Interstitial-lymphatic axis
(f) Circulatory switch
(g) Stem cancer cell

(ii) *Vitelline-like phenotype *
(a) Fat transport
(1) Lipoproteins
(2) Cholesterol
(b) Hematopoiesis
(1) Bone marrow control
(2) Platelets
(3) Neutrophils
(4) Mast cells
(5) Tumor-associated macrophages
(6) Lymphocytes
(7) Dendritic cells
(c) Lymphatic metastasis
(d) Immunotolerance
(e) Angiogenic switch
(f) Cachexia
(1) Using the host as vitellum

(iii) *Gastrulation–like phenotype *
(a) Tumoral stroma
(1) Epithelial-mesenchymal transition
(2) Granulation tissue
(b) Tumoral parenchyma
(1) Mesenchymal epithelial transition
(2) Tumor growth
(c) Tumoral organ
(1) Tumoral tissue heterogeneity

**Table 3:** Common metabolic and functional characteristics of the embryonic and the cancerous axis under hypoxia.

(i) Avascular stage of development
(ii) Notch-signaling pathway
(iii) Hedgehog-signaling pathway
(iv) Mammalian target of rapamycin (mTOR)
(v) Prolyl hydroxylase (PHD)-hypoxia-inducible transcription factor (HIF) system
(vi) Aerobic glycolysis (“Warburg effect”)
(vii) Oxidative and nitrosative stress
(viii) Antioxidant defenses: glutamine metabolism, oxidative and nonoxidative pentose phosphate pathways
(ix) Matrix metalloproteinases
(x) I*κ*B kinase/nuclear factor (NF)-*κ*B (IKK/NF-*κ*B) system
(xi) Chemokines and adhesion molecules
(xii) Toll-like receptors
(xiii) Lymphangiogenesis
(xiv) Prostaglandins and leukotrienes active metabolism
(xv) Mitogen-activated protein kinases (MAPKs)
(xvi) Tumoral/embryonic antigens
(xvii) Lymphoid neogenesis
(xviii) Epithelial mesenchymal transition
(xix) Mesenchymal stem cells/fibroblasts
(xx) Growth factors and chemotactic factors for mesenchymal stem cells
(a) Platelet-derived growth factor (PDGF)
(b) Transforming growth factor *β* (TGF-*β*)
(c) Fibroblast growth factor-2 (FGF-2)
(d) Hepatocyte growth factor (HGF)
(xxi) Angiogenesis (“angiogenic switch”)
(a) Vascular endothelial growth factor A (VEGF-A)
(b) Angiopoietin-1
(c) Endothelial cell chemotaxis
(xxii) Selective metabolic advantage

## Abbreviations

Angs: Angiopoietins
bFGF: Basic fibroblast growth factor
EMT: Epithelial to mesenchymal transitions
ERKs: Extracellular signal-regulated kinases
FGF-2: Fibroblast growth factor-2
HDL: High-density lipoproteins
HGF: Hepatocyte growth factor
HIF: Hypoxia-inducible transcription factorsystem
IKK: I*κ*B kinase
IKK/NF-*κ*B: I*κ*B kinase/Nuclear factor kappa B system
JNKs: c-Jun NH2-terminal kinases
MAPKs: Mitogen activated protein kinases
MET: Mesenchymal to epithelial transition
mTOR: Mammalian target of rapamycin
NF-*κ*B: Nuclear factor kappa B
PCI3K: Phosphoinositide 3-kinase
PDGF: Platelet-derived growth factor
PHDs: Prolyl hydroxylases
PTEN: Phosphatase and tensin homolog
Shh: Sonic hedgehog
SIRS: Systemic inflammatory response syndrome
TAMs: Infiltrating tumor associated macrophages
TGF-*β*: Transforming growth factor *β*
TLR: Toll-like receptors
VEGF-A: Vascular endothelial growth factor A
VLDL: Very- low-density lipoproteins.

## References

[1] Mantovani A, Allavena P, Sica A, Balkwill F (2008). Cancer-related inflammation. *Nature*.

[2] Dvorak HF (1986). Tumors: wounds that do not heal: similarities between tumor stroma generation and wound healing. *New England Journal of Medicine*.

[3] Moore MM, Chua W, Charles KA, Clarke SJ (2010). Inflammation and cancer: causes and consequences. *Clinical Pharmacology and Therapeutics*.

[4] Schäfer M, Werner S (2008). Cancer as an overhealing wound: an old hypothesis revisited. *Nature Reviews Molecular Cell Biology*.

[5] Aller MA, Arias JL, Nava MP, Arias J (2004). Posttraumatic inflammation is a complex response based on the pathological expression of the nervous, immune, and endocrine functional systems. *Experimental Biology and Medicine*.

[6] Aller MA, Arias JI, Giner M, Middleton JE (2011). Oxygen-related inflammatory wound phenotypes. *Wound Healing: Process, Phases and Promoting*.

[7] Aller MA, Arias JL, Sánchez-Patán F, Arias J (2006). The inflammatory response: an efficient way of life. *Medical Science Monitor*.

[8] Aller MA, Arias JI, Arias J (2010). Pathological axes of wound repair: gastrulation revisited. *Theoretical Biology and Medical Modelling*.

[9] De-Miguel MP, Arnalich-Montiel F, Lopez-Iglesias P, Blazquez-Martinez A, Nistal M (2009). Epiblast-derived stem cells in embryonic and adult tissues. *International Journal of Developmental Biology*.

[10] Bellini C, Boccardo F, Bonioli E, Campisi C (2006). Lymphodynamics in the fetus and newborn. *Lymphology*.

[11] Underwood MA, Gilbert WM, Sherman MP (2005). Amniotic fluid: not just fetal urine anymore. *Journal of Perinatology*.

[12] Kim BJ, Romero R, Lee SM (2011). Clinical significance of oligohydramnios in patients with preterm labor and intact membranes∗,∗∗. *Journal of Perinatal Medicine*.

[13] Parolini O, Soncini M, Evangelista M, Schmidt D (2009). Amniotic membrane and amniotic fluid-derived cells: potential tools for regenerative medicine?. *Regenerative Medicine*.

[14] Bielinska M, Narita N, Wilson DB (1999). Distinct roles for visceral endoderm during embryonic mouse development. *International Journal of Developmental Biology*.

[15] Collardeau-Frachon S, Scoazec JY (2008). Vascular development and differentiation during human liver organogenesis. *Anatomical Record*.

[16] Yoshida S, Wada Y (2005). Transfer of maternal cholesterol to embryo and fetus in pregnant mice. *Journal of Lipid Research*.

[17] Koike S, Keino-Masu K, Ohto T, Sugiyama F, Takahashi S, Masu M (2009). Autotaxin/lysophospholipase D-mediated lysophosphatidic acid signaling is required to form distinctive large lysosomes in the visceral endoderm cells of the mouse yolk Sac. *Journal of Biological Chemistry*.

[18] Terasawa Y, Cases SJ, Wong JS (1999). Apolipoprotein B-related gene expression and ultrastructural characteristics of lipoprotein secretion in mouse yolk sac during embryonic development. *Journal of Lipid Research*.

[19] Rantakari P, Lagerbohm H, Kaimainen M (2010). Hydroxysteroid (17*β*) dehydrogenase 12 is essential for mouse organogenesis and embryonic survival. *Endocrinology*.

[20] Arukwe A, Goksøyr A (2003). Eggshell and egg yolk proteins in fish: hepatic proteins for the next generation: oogenetic, population, and evolutionary implications of endocrine disruption. *Comparative Hepatology*.

[21] Sorrell JM, Caplan AI (2009). Fibroblasts-a diverse population at the center of it all. *International Review of Cell and Molecular Biology*.

[22] Hinz B (2010). The myofibroblast: paradigm for a mechanically active cell. *Journal of Biomechanics*.

[23] Pilling D, Fan T, Huang D, Kaul B, Gomer RH (2009). Identification of markers that distinguish monocyte-derived fibrocytes from monocytes, macrophages, and fibroblasts. *PLoS ONE*.

[24] Seta N, Kuwana M (2007). Human circulating monocytes as multipotential progenitors. *Keio Journal of Medicine*.

[25] Eckers B, Nischt R, Krieg T (2010). Cell-matrix interactions in dermal repair and scarring. *Fibrogenesis Tissue Repair*.

[26] Sen CK (2009). Wound healing essentials: let there be oxygen. *Wound Repair and Regeneration*.

[27] Chung HM, Won CH, Sung JH (2009). Responses of adipose-derived stem cells during hypoxia: enhanced skin-regenerative potential. *Expert Opinion on Biological Therapy*.

[28] Gurtner GC, Werner S, Barrandon Y, Longaker MT (2008). Wound repair and regeneration. *Nature*.

[29] Kalluri R, Weinberg RA (2009). The basics of epithelial-mesenchymal transition. *Journal of Clinical Investigation*.

[30] Selman M, Pardo A, Kaminski N (2008). Idiopathic pulmonary fibrosis: aberrant recapitulation of developmental programs?. *PLoS Medicine*.

[31] Whiteside TL (2008). The tumor microenvironment and its role in promoting tumor growth. *Oncogene*.

[32] Kluwe J, Mencin A, Schwabe RF (2009). Toll-like receptors, wound healing, and carcinogenesis. *Journal of Molecular Medicine*.

[33] Patel SA, Heinrich AC, Reddy BY, Rameshwar P (2009). Inflammatory mediators: parallels between cancer biology and stem cell therapy. *Journal of Inflammation Research*.

[34] Grivennikov SI, Greten FR, Karin M (2010). Immunity, inflammation, and cancer. *Cell*.

[35] Rodriguez-Vita J, Lawrence T (2010). The resolution of inflammation and cancer. *Cytokine and Growth Factor Reviews*.

[36] Schetter AJ, Heegaard NHH, Harris CC (2010). Inflammation and cancer: interweaving microRNA, free radical, cytokine and p53 pathways. *Carcinogenesis*.

[37] Allen M, Jones JL (2011). Jekyll and Hyde: the role of the microenvironment on the progression of cancer. *Journal of Pathology*.

[38] Costello LC, Franklin RB (2006). Tumor cell metabolism: the marriage of molecular genetics and proteomics with cellular intermediary metabolism; proceed with caution!. *Molecular Cancer*.

[39] Dalerba P, Cho RW, Clarke MF (2007). Cancer stem cells: models and concepts. *Annual Review of Medicine*.

[40] Visvader JE (2011). Cells of origin in cancer. *Nature*.

[41] DeBerardinis RJ, Lum JJ, Hatzivassiliou G, Thompson CB (2008). The biology of cancer: metabolic reprogramming fuels cell growth and proliferation. *Cell Metabolism*.

[42] Dang CV, Hamaker M, Sun P, Le A, Gao P (2011). Therapeutic targeting of cancer cell metabolism. *Journal of Molecular Medicine*.

[43] Shanware NP, Mullen AR, DeBerardinis RJ, Abraham RT (2011). Glutamine: pleiotropic roles in tumor growth and stress resistance. *Journal of Molecular Medicine*.

[44] Arias JI, Aller MA, Arias J (2005). The use of inflammation by tumor cells. *Cancer*.

[45] Arias JI, Aller MA, Sánchez-Patan F, Arias J (2006). Inflammation and cancer: is trophism the link?. *Surgical Oncology*.

[46] Arias JI, Aller MA, Arias J (2007). Cancer cell: using inflammation to invade the host. *Molecular Cancer*.

[47] Bishop A (2008). Role of oxygen in wound healing. *Journal of Wound Care*.

[48] Schreml S, Szeimies RM, Prantl L, Karrer S, Landthaler M, Babilas P (2010). Oxygen in acute and chronic wound healing. *British Journal of Dermatology*.

[49] Mareel M, Leroy A (2003). Clinical, cellular, and molecular aspects of cancer invasion. *Physiological Reviews*.

[50] Eltzschig HK, Carmeliet P (2011). Hypoxia and inflammation. *New England Journal of Medicine*.

[51] Bamias A, Dimopoulos MA (2003). Angiogenesis in human cancer: implications in cancer therapy. *European Journal of Internal Medicine*.

[52] Denko NC, Fontana LA, Hudson KM (2003). Investigating hypoxic tumor physiology through gene expression patterns. *Oncogene*.

[53] Heiden MGV, Cantley LC, Thompson CB (2009). Understanding the warburg effect: the metabolic requirements of cell proliferation. *Science*.

[54] Hornebeck W, Emonard H, Monboisse JC, Bellon G (2002). Matrix-directed regulation of pericellular proteolysis and tumor progression. *Seminars in Cancer Biology*.

[55] Gialeli C, Theocharis AD, Karamanos NK (2011). Roles of matrix metalloproteinases in cancer progression and their pharmacological targeting. *FEBS Journal*.

[56] Schmidt-Kittler O, Ragg T, Daskalakis A (2003). From latent disseminated cells to overt metastasis: genetic analysis of systemic breast cancer progression. *Proceedings of the National Academy of Sciences of the United States of America*.

[57] Freitas I, Baronzio GF, Bono B (1997). Tumor interstitial fluid: misconsidered component of the internal milieu of a solid tumor. *Anticancer Research*.

[58] Eisenhut M, Wallace H (2011). Ion channels in inflammation. *Pflugers Archiv European Journal of Physiology*.

[59] Wiig H, Tenstad O, Iversen PO, Kalluri R, Bjerkvig R (2010). Interstitial fluid: the overlooked component of the tumor microenvironment?. *Fibrogenesis and Tissue Repair*.

[60] Shieh AC, Swartz MA (2011). Regulation of tumor invasion by interstitial fluid flow. *Physical Biology*.

[61] Teng P-N, Hood BL, Sun M, Dhir R, Conrads TP (2011). Differential proteomic analysis of renal cell carcinoma tissue interstitial fluid. *Journal of Proteome Research*.

[62] Ronnov-Jessen L, Petersen OW, Bissell MJ (1996). Cellular changes involved in conversion of normal to malignant breast: importance of the stromal reaction. *Physiological Reviews*.

[63] Jiang D, Liang J, Noble PW (2007). Hyaluronan in tissue injury and repair. *Annual Review of Cell and Developmental Biology*.

[64] Chen B, Fu B A model for charged molecule transport in the interstitial space.

[65] Cueni LN, Detmar M (2008). The lymphatic system in health and disease. *Lymphatic Research and Biology*.

[66] Rutkowski JM, Swartz MA (2007). A driving force for change: interstitial flow as a morphoregulator. *Trends in Cell Biology*.

[67] Nagahashi M, Ramachandran S, Rashid OM, Takabe K (2010). Lymphangiogenesis: a new player in cancer progression. *World Journal of Gastroenterology*.

[68] Zhang Z, Helman JI, Li LJ (2010). Lymphangiogenesis, lymphatic endothelial cells and lymphatic metastasis in head and neck cancer–a review of mechanisms. *International Journal of Oral Science*.

[69] Lund AW, Swartz MA (2010). Role of lymphatic vessels in tumor immunity: passive conduits or active participants?. *Journal of Mammary Gland Biology and Neoplasia*.

[70] Al-Rawi MA, Jiang WG (2011). Lymphangiogenesis and cancer metastasis. *Frontiers in Bioscience*.

[71] Halliwell B (2007). Oxidative stress and cancer: have we moved forward?. *Biochemical Journal*.

[72] He G, Karin M (2011). NF-*κ*B and STAT3- key players in liver inflammation and cancer. *Cell Research*.

[73] Hanada T, Yoshimura A (2002). Regulation of cytokine signaling and inflammation. *Cytokine and Growth Factor Reviews*.

[74] Patel SA, Heinrich AC, Reddy BY, Rameshwar P (2009). Inflammatory mediators: parallels between cancer biology and stem cell therapy. *Journal of Inflammation Research*.

[75] Rakoff-Nahoum S, Medzhitov R (2009). Toll-like receptors and cancer. *Nature Reviews Cancer*.

[76] Wang D, Dubois RN (2010). Eicosanoids and cancer. *Nature Reviews Cancer*.

[77] Huang P, Han J, Hui L (2010). MAPK signaling in inflammation-associated cancer development. *Protein Cell*.

[78] Peyrou M, Bourgoin L, Foti M (2010). PTEN in liver diseases and cancer. *World Journal of Gastroenterology*.

[79] Philip M, Rowley DA, Schreiber H (2004). Inflammation as a tumor promoter in cancer induction. *Seminars in Cancer Biology*.

[80] Ben-Baruch A (2006). Inflammation-associated immune suppression in cancer: the roles played by cytokines, chemokines and additional mediators. *Seminars in Cancer Biology*.

[81] Nickoloff BJ, Ben-Neriah Y, Pikarsky E (2005). Inflammation and cancer: is the link as simple as we think?. *Journal of Investigative Dermatology*.

[82] Palucka K, Ueno H, Fay J, Banchereau J (2011). Dendritic cells and immunity against cancer. *Journal of Internal Medicine*.

[83] Mantovani A, Sica A (2010). Macrophages, innate immunity and cancer: balance, tolerance, and diversity. *Current Opinion in Immunology*.

[84] Balkwill F, Mantovani A (2010). Cancer and inflammation: implications for pharmacology and therapeutics. *Clinical Pharmacology and Therapeutics*.

[85] Mishra P, Banerjee D, Ben-Baruch A (2010). Chemokines at the crossroads of tumor-fibroblast interactions that promote malignancy. *Journal of Leukocyte Biology*.

[86] Sica A (2010). Role of tumour-associated macrophages in cancer-related inflammation. *Experimental Oncology*.

[87] Chetty C, Rao JS, Lakka SS (2011). Matrix metalloproteinase pharmacogenomics in non-small-cell lung carcinoma. *Pharmacogenomics*.

[88] Engbring JA, Kleinman HK (2003). The basement membrane matrix in malignancy. *Journal of Pathology*.

[89] Müller A, Homey B, Soto H (2001). Involvement of chemokine receptors in breast cancer metastasis. *Nature*.

[90] Al-Rawi MAA, Mansel RE, Jiang WG (2005). Lymphangiogenesis and its role in cancer. *Histology and Histopathology*.

[91] Kraljevic Pavelic S, Sedic M, Bosnjak H, Spaventi S, Pavelic K (2011). Metastasis: new perspectives on an old problem. *Molecular Cancer*.

[92] Sitkovsky MV, Lukashev D, Apasov S (2004). Physiological control of immune response and inflammatory tissue damage by hypoxia-inducible factors and adenosine A2A receptors. *Annual Review of Immunology*.

[93] Levine AJ, Puzio-Kuter AM (2010). The control of the metabolic switch in cancers by oncogenes and tumor suppressor genes. *Science*.

[94] Mentis A-FA, Kararizou E (2010). Metabolism and cancer: an up-to-date review of a mutual connection. *Asian Pacific Journal of Cancer Prevention*.

[95] Naresh KN, Nerurkar AY, Borges AM (2001). Angiogenesis is redundant for tumour growth in lymph node metastases. *Histopathology*.

[96] Sleeman JP, Nazarenko I, Thiele W (2011). Do all roads lead to Rome? Routes to metastasis development. *International Journal of Cancer*.

[97] Chouaib S, Kieda C, Benlalam H, Noman MZ, Mami-Chouaib F, Rüegg C (2010). Endothelial cells as key determinants of the tumor microenvironment: interaction with tumor cells, extracellular matrix and immune killer cells. *Critical Reviews in Immunology*.

[98] Sleeman JP, Thiele W (2009). Tumor metastasis and the lymphatic vasculature. *International Journal of Cancer*.

[99] Ji R-C (2009). Lymph node lymphangiogenesis: a new concept for modulating tumor metastasis and inflammatory process. *Histology and Histopathology*.

[100] Pantel K, Woelfle U (2005). Detection and molecular characterisation of disseminated tumor cells: implications for anticancer therapy. *Biochimica et Biophysica Acta*.

[101] Braun S, Kentenich C, Janni W (2000). Lack of effect of adjuvant chemotherapy on the elimination of single dormant tumor cells in bone marrow of high-risk breast cancer patients. *Journal of Clinical Oncology*.

[102] Tammela T, Saaristo A, Holopainen T (2011). Photodynamic ablation of lymphatic vessels and intralymphatic cancer cells prevents metastasis. *Science Translational Medicine*.

[103] Deans C, Wigmore SJ (2005). Systemic inflammation, cachexia and prognosis in patients with cancer. *Current Opinion in Clinical Nutrition and Metabolic Care*.

[104] Delano MJ, Moldawer LL (2006). The origin of cachexia in acute and chronic inflammatory diseases. *Nutrition in Clinical Practice*.

[105] Fearon K, Strasser F, Anker SD (2011). Definition and classification of cancer cachexia: an international consensus. *The Lancet Oncology*.

[106] Clement CC, Rotzschke O, Santambrogio L (2011). The lymph as a pool of self-antigens. *Trends in Immunology*.

[107] Bruyère F, Noël A (2010). Lymphangiogenesis: in vitro and in vivo models. *FASEB Journal*.

[108] Thaunat O, Kerjaschki D, Nicoletti A (2006). Is defective lymphatic drainage a trigger for lymphoid neogenesis?. *Trends in Immunology*.

[109] Condamine T, Gabrilovich DI (2011). Molecular mechanisms regulating myeloid-derived suppressor cell differentiation and function. *Trends in Immunology*.

[110] Delavary BM, van der Veer WM, van Egmond M, Niessen FB, Beelen RHJ (2011). Macrophages in skin injury and repair. *Immunobiology*.

[111] Goldberg SR, Diegelmann RF (2010). Wound healing primer. *Surgical Clinics of North America*.

[112] Shih B, Garside E, McGrouther DA, Bayat A (2010). Molecular dissection of abnormal wound healing processes resulting in keloid disease. *Wound Repair and Regeneration*.

[113] Wu Y, Zhao RCH, Tredget EE (2010). Concise review: bone marrow-derived stem/progenitor cells in cutaneous repair and regeneration. *Stem Cells*.

[114] Acloque H, Adams MS, Fishwick K, Bronner-Fraser M, Nieto MA (2009). Epithelial-mesenchymal transitions: the importance of changing cell state in development and disease. *Journal of Clinical Investigation*.

[115] Sioud M (2011). New insights into mesenchymal stromal cell-mediated T-cell suppression through galectins. *Scandinavian Journal of Immunology*.

[116] Norrby K (2006). In vivo models of angiogenesis. *Journal of Cellular and Molecular Medicine*.

[117] Fukumura D, Duda DG, Munn LL, Jain RK (2010). Tumor microvasculature and microenvironment: novel insights through intravital imaging in pre-clinical models. *Microcirculation*.

[118] Van Horssen R, Ten Hagen TLM, Eggermont AMM (2006). TNF-*α* in cancer treatment: molecular insights, antitumor effects, and clinical utility. *Oncologist*.

[119] Byrne AM, Bouchier-Hayes DJ, Harmey JH (2005). Angiogenic and cell survival functions of Vascular Endothelial Growth Factor (VEGF). *Journal of Cellular and Molecular Medicine*.

[120] Kalluri R, Zeisberg M (2006). Fibroblasts in cancer. *Nature Reviews Cancer*.

[121] Grigorova IL, Panteleev M, Cyster JG (2010). Lymph node cortical sinus organization and relationship to lymphocyte egress dynamics and antigen exposure. *Proceedings of the National Academy of Sciences of the United States of America*.

[122] Hayasaka H, Taniguchi K, Fukai S, Miyasaka M (2010). Neogenesis and development of the high endothelial venules that mediate lymphocyte trafficking. *Cancer Science*.

[123] Bao X, Moseman EA, Saito H (2010). Endothelial heparan sulfate controls chemokine presentation in recruitment of lymphocytes and dendritic cells to lymph nodes. *Immunity*.

[124] Favaro E, Amadori A, Indraccolo S (2008). Cellular interactions in the vascular niche: implications in the regulation of tumor dormancy. *Acta Pathologica, Microbiologica et Immunologica Scandinavica*.

[125] Pinto M, Soares P, Ribatti D (2011). Thyroid hormone as a regulator of tumor induced angiogenesis. *Cancer Letters*.

[126] Hanahan D, Weinberg RA (2011). Hallmarks of cancer: the next generation. *Cell*.

[127] Cairns RA, Harris IS, Mak TW (2011). Regulation of cancer cell metabolism. *Nature Reviews Cancer*.

[128] Nakamura H, Hase A (1990). Cellular differentiation in the process of generation of the eukaryotic cell. *Origins of Life and Evolution of the Biosphere*.

[129] Goldsmith P, Fenton H, Morris-Stiff G, Ahmad N, Fisher J, Prasad KR (2010). Metabonomics: a useful tool for the future surgeon. *Journal of Surgical Research*.

[130] Serkova NJ, Spratlin JL, Eckhardt SG (2007). NMR-based metabolomics: translational application and treatment of cancer. *Current Opinion in Molecular Therapeutics*.

[131] Herling A, König M, Bulik S, Holzhütter HG (2011). Enzymatic features of the glucose metabolism in tumor cells. *FEBS Journal*.

[132] Brahimi-Horn MC, Chiche J, Pouysségur J (2007). Hypoxia and cancer. *Journal of Molecular Medicine*.

[133] Matsumoto S, Yasui H, Mitchell JB, Krishna MC (2010). Imaging cycling tumor hypoxia. *Cancer Research*.

[134] De Filippis L, Delia D (2011). Hypoxia in the regulation of neural stem cells. *Cellular and Molecular Life Sciences*.

[135] Stockhausen MT, Kristoffersen K, Poulsen HS (2010). The functional role of Notch signaling in human gliomas. *Neuro-Oncology*.

[136] Pierfelice T, Alberi L, Gaiano N (2011). Notch in the vertebrate nervous system: an old dog with new tricks. *Neuron*.

[137] Subramaniam D, Ramalingam S, Houchen CW, Anant S (2010). Cancer stem cells: a novel paradigm for cancer prevention and treatment. *Mini Reviews in Medicinal Chemistry*.

[138] Guo S, Liu M, Gonzalez-Perez RR (2011). Role of Notch and its oncogenic signaling crosstalk in breast cancer. *Biochimica et Biophysica Acta—Reviews on Cancer*.

[139] Takebe N, Harris PJ, Warren RQ, Ivy SP (2011). Targeting cancer stem cells by inhibiting Wnt, Notch, and Hedgehog pathways. *Nature Reviews Clinical Oncology*.

[140] Bartkowiak K, Riethdorf S, Pantel K The interrelating dynamics of hypoxic tumor microenvironments and cancer cell phenotypes in cancer metastasis.

[141] Kruger NJ, Von Schaewen A (2003). The oxidative pentose phosphate pathway: structure and organisation. *Current Opinion in Plant Biology*.

[142] Krüger A, Ralser M (2011). ATM is a redox sensor linking genome stability and carbon metabolism. *Science Signaling*.

[143] Yecies JL, Manning BD (2011). MTOR links oncogenic signaling to tumor cell metabolism. *Journal of Molecular Medicine*.

[144] Perl A, Hanczko R, Telarico T, Oaks Z, Landas S (2011). Oxidative stress, inflammation and carcinogenesis are controlled through the pentose phosphate pathway by transaldolase. *Trends in Molecular Medicine*.

[145] Gibbons JJ, Abraham RT, Yu K (2009). Mammalian target of rapamycin: discovery of rapamycin reveals a signaling pathway important for normal and cancer cell growth. *Seminars in Oncology*.

[146] Yecies JL, Manning BD (2011). Transcriptional control of cellular metabolism by mtor signaling. *Cancer Research*.

[147] Zhang K, Zhu L, Fan M (2011). Oxygen, a key factor regulating cell behavior during neurogenesis and cerebral diseases. *Frontiers in Molecular Neuroscience*.

[148] Kume T (2010). Specification of arterial, venous, and lymphatic endothelial cells during embryonic development. *Histology and Histopathology*.

[149] Morrow D, Cullen JP, Liu W (2009). Sonic hedgehog induces notch target gene expression in vascular smooth muscle cells via VEGF-A. *Arteriosclerosis, Thrombosis, and Vascular Biology*.

[150] Onimaru M, Yonemitsu Y (2011). Angiogenic and lymphangiogenic cascades in the tumor microenvironment. *Frontiers in Bioscience*.

[151] Takenaga K (2011). Angiogenic signaling aberrantly induced by tumor hypoxia. *Frontiers in Bioscience*.

[152] Burger RA (2011). Overview of anti-angiogenic agents in development for ovarian cancer. *Gynecologic Oncology*.

[153] Rodríguez-Enríquez S, Gallardo-Pérez JC, Marín-Hernández A (2011). Oxidative phosphorylation as a target to arrest malignant neoplasias. *Current Medicinal Chemistry*.

[154] Israël M, Schwartz L (2011). The metabolic advantage of tumor cells. *Molecular Cancer*.

[155] Adinolfi A, Adinolfi M, Lessof MH (1975). Alpha feto protein during development and in disease. *Journal of Medical Genetics*.

[156] Sell S, Pierce GB (1994). Maturation arrest of stem cell differentiation is a common pathway for the cellular origin of teratocarcinomas and epithelial cancers. *Laboratory Investigation*.

[157] Brewer BG, Mitchell RA, Harandi A, Eaton JW (2009). Embryonic vaccines against cancer: an early history. *Experimental and Molecular Pathology*.

[158] Hendrix MJC, Seftor EA, Seftor REB, Kasemeier-Kulesa J, Kulesa PM, Postovit LM (2007). Reprogramming metastatic tumour cells with embryonic microenvironments. *Nature Reviews Cancer*.

[159] Strizzi L, Hardy KM, Kirsammer GT, Gerami P, Hendrix MJC (2011). Embryonic signaling in melanoma: potential for diagnosis and therapy. *Laboratory Investigation*.

[160] Diegelmann RF, Evans MC (2004). Wound healing: an overview of acute, fibrotic and delayed healing. *Frontiers in Bioscience*.

[161] Detoraki A, Granata F, Staibano S, Rossi FW, Marone G, Genovese A (2010). Angiogenesis and lymphangiogenesis in bronchial asthma. *Allergy*.

[162] Klueh U, Kaur M, Qiao Y, Kreutzer DL (2010). Critical role of tissue mast cells in controlling long-term glucose sensor function in vivo. *Biomaterials*.

[163] Van Der Veer WM, Bloemen MC, Ulrich MM (2009). Potential cellular and molecular causes of hypertrophic scar formation. *Burns*.

[164] Dazzi F, Krampera M (2011). Mesenchymal stem cells and autoimmune diseases. *Best Practice and Research: Clinical Haematology*.

[165] Yang L, Pang Y, Moses HL (2010). TGF-*β* and immune cells: an important regulatory axis in the tumor microenvironment and progression. *Trends in Immunology*.

[166] Neurath MF, Finotto S (2011). IL-6 signaling in autoimmunity, chronic inflammation and inflammation-associated cancer. *Cytokine and Growth Factor Reviews*.

[167] Campbell I, Qiu W, Haviv I (2011). Genetic changes in tumour microenvironments. *Journal of Pathology*.

[168] da Silva Meirelles L, Fontes AM, Covas DT, Caplan AI (2009). Mechanisms involved in the therapeutic properties of mesenchymal stem cells. *Cytokine and Growth Factor Reviews*.

[169] Roh TS, Rah DK, Park BY (2001). The fetal wound healing: a review. *Yonsei Medical Journal*.

[170] Larson BJ, Longaker MT, Lorenz HP (2010). Scarless fetal wound healing: a basic science review. *Plastic and Reconstructive Surgery*.

[171] Thiery JP, Acloque H, Huang RYJ, Nieto MA (2009). Epithelial-mesenchymal transitions in development and disease. *Cell*.

[172] Singh A, Settleman J (2010). EMT, cancer stem cells and drug resistance: an emerging axis of evil in the war on cancer. *Oncogene*.

[173] Wang Y, Steinbeisser H (2009). Molecular basis of morphogenesis during vertebrate gastrulation. *Cellular and Molecular Life Sciences*.

[174] Simon MC, Keith B (2008). The role of oxygen availability in embryonic development and stem cell function. *Nature Reviews Molecular Cell Biology*.

[175] Chang YJ, Hwang SM, Tseng CP (2010). Isolation of mesenchymal stem cells with neurogenic potential from the mesoderm of the amniotic membrane. *Cells Tissues Organs*.

[176] Pfeiffer S, McLaughlin D (2010). In vitro differentiation of human amniotic fluid-derived cells: augmentation towards a neuronal dopaminergic phenotype. *Cell Biology International*.

[177] Niknejad H, Peirovi H, Jorjani M, Ahmadiani A, Ghanavi J, Seifalian AM (2008). Properties of the amniotic membrane for potential use in tissue engineering. *European Cells and Materials*.

[178] Seong JY, Soncini M, Kaneko Y, Hess DC, Parolini O, Borlongan CV (2009). Amnion: a potent graft source for cell therapy in stroke. *Cell Transplantation*.

[179] Uberti MG, Pierpont YN, Ko F (2010). Amnion-derived cellular cytokine solution (ACCS) promotes migration of keratinocytes and fibroblasts. *Annals of Plastic Surgery*.

[180] Gasperowicz M, Natale RR (2009). Establishing Three Blastocyst Lineages—Then What?. *Biology of Reproduction*.

[181] Fraser ST, Baron MH (2009). Embryonic fates for extraembryonic lineages: new perspectives. *Journal of Cellular Biochemistry*.

[182] Sheng G (2010). Primitive and definitive erythropoiesis in the yolk sac: a bird’s eye view. *International Journal of Developmental Biology*.

[183] Lander ES (2011). Initial impact of the sequencing of the human genome. *Nature*.

[184] Pérez-Losada J, Castellanos-Martín A, Mao J-H (2011). Cancer evolution and individual susceptibility. *Integrative Biology*.

[185] Stratton MR, Campbell PJ, Futreal PA (2009). The cancer genome. *Nature*.

